# Mental Health of Healthcare Professionals: Two Years of the COVID-19 Pandemic in Portugal

**DOI:** 10.3390/ijerph20043131

**Published:** 2023-02-10

**Authors:** Alexandra Costa, Teresa Caldas de Almeida, Mónica Fialho, Célia Rasga, Hugo Martiniano, Osvaldo Santos, Ana Virgolino, Astrid Moura Vicente, Maria João Heitor

**Affiliations:** 1 Departamento de Promoção da Saúde e Prevenção de Doenças Não-Transmissíveis, Instituto Nacional de Saúde Doutor Ricardo Jorge, 1649-016 Lisboa, Portugal; 2 Environmental Health Behaviour Lab, Instituto de Saúde Ambiental, Faculdade de Medicina, Universidade de Lisboa, 2649-028 Lisboa, Portugal; 3 Laboratório Associado TERRA, Faculdade de Medicina, Universidade de Lisboa, 2649-028 Lisboa, Portugal; 4 BioISI–Biosystems and Integrative Sciences Institute, Faculdade de Ciências, Universidade de Lisboa, 1749-016 Lisboa, Portugal; 5 Unbreakable Idea Research, 2550-426 Painho, Portugal; 6 Departamento de Psiquiatria e Saúde Mental, Hospital Beatriz Ângelo, 2674-514 Loures, Portugal; 7 Sociedade Portuguesa de Psiquiatria e Saúde Mental, 1050-096 Lisboa, Portugal; 8 Centro de Investigação Interdisciplinar em Saúde (CIIS), Faculdade de Medicina, Universidade Católica Portuguesa, 2635-631 Sintra, Portugal

**Keywords:** anxiety, depression, stress disorders, post-traumatic, professional burnout, resilience, mental health promotion, risk and protective factors

## Abstract

The COVID-19 pandemic increased psychosocial risk factors among healthcare professionals (HCPs). Objective: To characterize Portuguese HCPs mental health (MH), estimate anxiety, depression, post-traumatic stress disorder (PTSD) and burnout symptoms, and identify risk/protective factors. A cross-sectional online survey and a longitudinal assessment were conducted in 2020 (T0) and 2021 (T1). Sociodemographic and occupational variables, COVID-19-related experiences and protective behavior data were collected from a non-probabilistic sample of HCPs in Portugal. Symptoms of anxiety, depression, PTSD, burnout and resilience were assessed using the Portuguese versions of the Generalized Anxiety Disorder Scale (GAD-7), the Patient Health Questionnaire (PHQ-9), the Post-traumatic Stress Disorder Checklist (PCL-5), the Shirom–Melamed Burnout Measure (MBSM) and the Connor–Davidson Resilience Scale (CD-RISC-10), respectively. Risk and protective factors were identified through simple and multiple logistic regression models. Overall, 2027 participants answered the survey in T0 and 1843 in T1. The percentage of moderate-to-severe symptoms decreased from T0 to T1; however, a considerable proportion of HCPs reported symptoms of distress in both years. Being a woman, working in a COVID-19-treatment frontline position and work–life balance increased the odds of distress. High resilience, good social/family support, and hobbies/lifestyle maintenance were found to be protective factors. Globally, our results show that performing as a HCP during the pandemic may result in long-term effects on MH.

## 1. Introduction

In the face of a pandemic, a health system operating at maximum capacity is essential. To achieve this, healthcare professionals (HCPs) are the most valuable resource. According to the last European Working Conditions Survey, workers from the health sector are exposed to the highest levels of work intensity, which includes aspects related to working at high speed and under time pressure, as well as experiencing high emotional demands [1]. Evidence indicates that high levels of emotional demands are linked to mental health (MH) problems, such as depression, anxiety, long-term sickness absence, fatigue and burnout [1,2]. The COVID-19 pandemic has aggravated and multiplied the presence of these pre-existing psychosocial risk factors for HCP wellbeing [3].

Unlike the general population, HCPs were called to the frontline, diagnosing, treating and taking care of COVID-19 patients. These professionals have played a crucial role during this public health crisis, exposing themselves to both physical and psychological hazards [4,5]. The pandemic has placed an extraordinary psychological and emotional burden on HCPs: performing in high-demanding settings, overworked, afraid of exposing themselves, their colleagues and their families to the virus and reducing/avoiding contact with their families and friends. Due to these contextual changes, particularly work-overload and social isolation, there was a significant reduction and deterioration in their usual sources of social support [6,7]. The loss of familiar and social support, an increased work overload and emotional demand experienced during the pandemic could have compromised their individual resilience and coping skills [8]. Resilience can be understood as flexible individual adaptation to stressful events and enhanced recovery from negative experiences [9]. Furthermore, low resilience level can be a factor contributing to psychological distress.

Distress can be multifactorial and may affect wellbeing, work performance and efficacy. Mental health complaints among HCPs have emerged within the context of COVID-19 with significant worldwide self-reported symptoms of anxiety, depression, PTSD and burnout (e.g., [10,11,12,13,14,15]). Systematic reviews identified COVID-19-related risk factors for physical and mental wellbeing, such as increased workload, physical exhaustion, inadequate personal protective equipment, exposure to patients with COVID-19, the fear of infecting others and the need to make ethically difficult decisions, as major contributors to HCPs’ reported distress [16,17,18]. Additionally, working with new and frequently changing protocols and the lack of expertise in treating COVID-19 patients could be related to increased distress [19,20].

Furthermore, the meta-analysis of Pappa et al. [16] revealed potentially relevant gender and occupational differences for reported symptoms of anxiety and depression, with women and nursing staff showing higher percentages than their counterparts. Moreover, according to the review performed by Muller et al. [17], the most common factors correlated with increased risk of distress was the exposure to COVID-19 patients, being a woman and worry about infection of oneself or family members.

Inversely, the most reported MH protective factors were the perception of having available social support [8,21] and a higher resilience level [8,22,23]. Both resilience and social support have been shown to be psychological buffers in periods of demanding stress and act as protective factors against burnout and post-traumatic stress disorder symptoms [9]. In the specific context of COVID-19 pandemic, research demonstrates that increased resilience is linked to increased reported wellbeing [22,24].

While some cross-sectional evidence on HCP mental health during the COVID-19 pandemic is available, longitudinal analysis reflecting within-person changes of mental health outcomes is still scarce [25]. Sound evidence is required, not only to assess distress among HCPs, but to identify risk factors and occupational stressors, supporting the design of evidence-based primary prevention strategies and the adoption of mitigation actions (secondary prevention) [26].

Understanding the psychological needs of HCPs should be prioritized to provide them with the appropriate tools to identify and mitigate MH impairments, to improve HCPs’ mental health literacy through training and enhance coping skills and to better manage occupational stress and strengthen individual resilience, can be complementary approaches to protect HCP mental health and, consequently, contribute to the systemic efforts needed to fight a public health crisis [9,27]. Moreover, special attention should be given to work–life balance promotion and additional organizational measures should be adopted to address it. The successful management of stressors can have a protective effect in future crises.

Portuguese data around the psychological burden of the COVID-19 pandemic on HCPs are still scarce. The existing evidence is mainly concerned with the general population, targeting sociodemographic determinants of mental health [28,29,30]. To address this gap, this research focuses on HCP MH outcomes in Portugal, with a longitudinal perspective, whilst still exploring the role of occupational and COVID-19 contextual risk and protective factors.

The main goal of this study was to characterize Portuguese HCPs’ MH and to identify risk/protective factors and their association with reported psychological symptoms in 2020 and 2021. For this purpose, we estimated the percentage of symptoms of anxiety, depression, post-traumatic stress disorder (PTSD) and burnout according to sociodemographic (i.e., age, gender, etc.) and occupational characteristics (i.e., working position, workload, etc.) and a number of factors, such as COVID-19-related experiences and protective behaviors.

## 2. Materials and Methods

### 2.1. Study Design and Participants

The study reported here is part of a broader project, the SM-COVID19 Project (Saúde Mental em Tempos de Pandemia COVID-19|Mental Health during the COVID-19 Pandemic), aimed at characterizing the MH and wellbeing of the adult general population and HCPs (data provided here) residing in Portugal during the first two years of the COVID-19 pandemic (https://sm-covid19.pt/ accessed on 16 January 2023).

A cross-sectional study with a longitudinal component was designed to assess the psychological responses of HCPs and related risk and protective factors during a two-year period of the COVID-19 pandemic in Portugal. A self-administered online survey was conducted via the Limesurvey^®^ platform. The SM-COVID19 survey is available in Portuguese as Supplementary material. A convenience sample of HCPs working across Portugal with different professional and clinical careers (physicians, nurses, healthcare assistants and others, such as diagnostic therapists, pharmaceutics, etc.), settings (hospitals, primary care units or nursing homes; frontline and non-frontline) and activity sectors (public, private and social) was invited to participate in the study. Invitations were sent via email to health institutional leaders and professional associations, with a request to circulate to staff/members using a snowball approach.

Data collection took place in two moments between May–July 2020 (T0) and May–July 2021 (T1). All respondents provided informed consent at the beginning of the survey confirming their willingness to participate in the study voluntarily. Those who had explicitly consented at T0 to be surveyed in a posterior data collection, by means of the contact details provided by them (email address), were provided with a survey link to answer the questionnaire at T1.

### 2.2. Ethical Approval and Consent

Ethical approval was granted by the Ethical Committee for Health from the National Institute of Health Doutor Ricardo Jorge, I.P. (reference number: 119-2020). This Committee addressed a set of measures to guarantee compliance with ethical procedures for data collection and data management. The study was conducted in accordance with the Declaration of Helsinki. Written online informed consent was obtained from all participants.

### 2.3. Inclusion/Exclusion Criteria

Respondents were included in this study if they had a cumulative age ≥18–90 years and self-identified as HCPs.

Exclusion criteria were (a) duplicated questionnaires regarding answers on sex, birthdate and postal code; and (b) meaningless data based on short response time and/or repetitive patterns (e.g., answers given to Likert-type scales), according to Leiner [31].

### 2.4. Outcomes and Covariables

#### 2.4.1. Mental Health Outcomes

The key mental health outcomes selected were symptoms of anxiety, depression, post-traumatic stress disorder (PTSD) and burnout, as well as resilience. To assess these outcomes, a set of instruments with sound psychometric properties were used and their internal consistency was calculated with Cronbach’s α.

Anxiety was assessed using the Portuguese version of the Generalized Anxiety Disorder Scale (GAD-7) [32]. This scale has a one-dimensional structure, consisting of 7 items which correspond to the DSM-IV diagnostic criteria. Each item is rated in a 4-point Likert type scale, ranging from 0 (“not at all”) to 4 (“nearly every day”). The total score of the GAD-7 ranges from 0 to 21 points, with the following cut-off scores: 0–9 points—missing-to-mild anxiety symptoms and 10–21 points—moderate-to-severe anxiety symptoms. Within this sample, Cronbach’s α for this scale was 0.91.

The translated and validated version of the Patient Health Questionnaire (PHQ-9) [33] was used to assess depression. This scale includes 9 items, each one rated in a 4-point Likert type scale, ranging from 0 (“not at all”) to 4 (“nearly every day”). The total score of the PHQ-9 ranges from 0 to 27 points, with the following cut-off scores: 0–9 points—missing-to-mild symptoms and 10–27 points—moderate-to-severe symptoms (Cronbach’s α = 0.88).

Post-traumatic stress disorder (PTSD) was assessed using the Short Form of the Post-traumatic Stress Disorder Checklist (PCL-5) for DSM-V [34]. PCL-5 Short Form is a 4-item scale developed for evaluating the presence and severity of PTSD symptoms according to DSM-V criteria. Respondents rated the degree to which they were affected by each symptom in a 5-point Likert type scale, ranging from 0 (“not at all”) to 5 (“extremely”). Since no adapted and validated version of PCL-5 Short Form exists for the Portuguese population, two members of the research team independently translated the scale; the research team appraised the two versions and agreed on the final version to be used in the survey. Although the original scale asks respondents to consider the previous month, given the pandemic timeframe in T0 and to keep consistency across scales, respondents were instructed to refer to the two previous weeks. The final score is obtained by adding the scores of each item. Considered score ranges of 7–16 points indicate the presence of symptoms; 0–6 points indicate no symptoms. Within this sample, Cronbach’s α was 0.79 (T0) and 0.81 (T1).

Burnout assessment was performed using the Shirom–Melamed Burnout Measure (MBSM) [35,36]. This 14-item scale is organized into 3 subscales which correspond to physical fatigue (6 items), cognitive fatigue (5 items) and emotional exhaustion (3 items) dimensions. More specifically, participants were asked how many times in the last month they have had a certain feeling or difficulty in relation to their work (e.g., tiredness, fatigue, difficulty concentrating and interpersonal relationships), positioning themselves on a 7-point Likert scale, where 1 is “Never or almost never” and 7 “Always or almost always”. According to the total scale score, the following classification was adopted: 1 to 3 points—no burnout; 4 to 7 points—burnout (Cronbach’s α = 0.96).

Lastly, to assess participants’ resilience levels, a briefer version of the 10-item Connor–Davidson Resilience Scale (CD-RISC-10) validated for the Portuguese population was chosen, which measures individual coping skills and health needs [37]. More specifically, participants were asked to position themselves according to their agreement with the 10 items, using a 5-point Likert scale (1 means “Nothing true” and 5 “Almost always true”). In the absence of a pre-established cut-off point, a three-level category classification was adopted. These levels were defined through cluster analysis (k-means; with bootstrap, 100 replications) according to the total score obtained in the scale: low (0–22 in 2020; 0–20 in 2021), medium (23–30 in 2020; 21–29 in 2021) and high (31–40 in 2020; 30–40 in 2021). For analysis, low and medium levels were grouped and compared against high levels of resilience. The Portuguese version of CD-RISC10 has shown good reliability and validity [37]. Within this sample, Cronbach’s α was 0.90.

All the above-described instruments consist of screening tools with sound psychometric properties, but they do not translate nor substitute a clinical diagnosis.

#### 2.4.2. Sociodemographic and Occupational-Related Data

Sociodemographic and occupational characteristics were collected. Variables assessed included sex, geographic location, educational level, income, professional career, professional setting (i.e., hospital, primary care center, nursing home), sector of activity (i.e., public, private, social), workload and direct contact with COVID-19 patients. For data analysis, geographic location was codified accordingly with the Nomenclature of Territorial Units for Statistics (NUTS) system for Portugal, subdivision II. Determination of frontline workers (defined as direct contact with COVID-19-infected patients) were obtained with self-definition through a binary question “Are you currently at the frontline dealing with COVID-19 patients?”.

#### 2.4.3. COVID-19-Related Experiences and Protective Behaviors

To detect contextual and occupational perceptions and pandemic-related concerns, the following survey items were selected: familiar adjustments (e.g., the need to move away from family and perceived work/family reconciliation); perceived social/family support; and contextual behavioral indicators, such as the maintenance of hobbies and lifestyle.

The variables of work–life balance, social/family support and hobbies and lifestyle were constructs obtained from multiple items of the questionnaire, developed by the research team and assessed on a 5-point Likert scale from 1 (“I totally disagree”) to 5 (“I totally agree”), plus a “non-applicable” (treated as missing). The agreement of the participants with each of the following sentences were considered for:

**Work–life balance**: ‘I have been able to reconcile work and household tasks’; ‘I have been able to reconcile work and childcare responsibilities (children aged < 6 years)’; ‘I have been able to reconcile work and providing school support to the children I take care of (children aged 6 to 10 years)’; ‘I have been able to reconcile work and providing school support to the youngsters I take care of (children aged 11 to 17 years)’; ‘I have been working late or during the weekends more often than I used to before the pandemic’; and ‘My work has been interfering with my personal and family life more often than it did before the pandemic’.

**Social/family support:** (Four items retrieved from the Brief Form of the Perceived Social Support Questionnaire (F-SozU K-6) [38]: ‘I experience a lot of understanding and security from others’; ‘I know a very close person whose help I can always count on’; ‘When I am sick, I can without hesitation ask friends and family to take care of important matters for me’; and ‘If I am down, I know to whom I can go without hesitation’.

**Hobbies and lifestyle:** ‘I can maintain my usual hobbies/hobbies’; ‘I can maintain a daily routine (e.g., wake up and bedtime, meals, work)’; ‘The physical activity I am doing is important to me’; ‘The help I am giving to other people (volunteering or otherwise) is important to me’; ‘Watching cultural events online (e.g., theater piece, musical show) is important to me’; ‘Reading is important to me (books, magazines, newspapers)’; ‘Looking for information and seeing news about the pandemic is important to me’; ‘I find myself looking for information and watching news about the pandemic throughout the day’; ‘Watching television, movies, series, documentaries is important to me’; ‘Doing gardening or other manual work is important to me’; ‘Playing games (video games, board games, crossword puzzles, sudoku) is important to me’; ‘It is difficult for me to have left my religious practice in the community’; and ‘It is difficult for me to have stopped going to cafes, restaurants, shopping and other activities (e.g., walking, travel)’.

For the purpose of data analysis, some of the above items were reversed (first four items for work–life balance; all six items for social and family support; items 1–7 and 9–11 for hobbies and lifestyle) and an average value was calculated for each individual on a scale of 1 to 5 (from best to worst situation). Then, a cut-off value of 2.5 was set, with lower values representing positive outcomes (e.g., ‘No difficulties in work–life balance’).

### 2.5. Statistical Analysis

Answers given to open-ended questions were analyzed and recoded as appropriate for further analysis, including professional career. In this regard, the participants were grouped into four categories: physicians, nurses, healthcare assistants and others. Included in the latter group were hospital administrators, pharmacists, and other health professional careers (e.g., diagnostic and therapeutic technicians, health technicians, etc.). Regarding workload, individuals that increased their number of work hours were compared against the ones that maintained or reduced them. For other questions, the following answers were treated as missing data (for the respective variables): (a) invalid postal codes (region), (b) “I rather not to respond” (monthly income) and (c) “non-applicable” (working position; needed to move away from family residency).

In the sample characterization, absolute and relative frequencies are presented for sociodemographic and occupational variables both in 2020 and in 2021.

Absolute and relative frequency (%) and respective 95% confidence interval (CI) of moderate-to-severe symptoms of anxiety (hereafter referred to as anxiety), depression (hereafter referred to as depression), post-traumatic stress disorder and burnout were estimated for each year independently. Bivariate analyses were conducted to explore the associations between these outcomes and sociodemographic and occupational variables, protection behaviors and pandemic-context data by using Pearson’s chi-square tests with second-order Rao–Scott correction.

To identify potential risk and protective factors for symptoms of psychological distress (anxiety, depression, post-traumatic stress disorder and burnout) in each year, simple and multiple logistic regression models were applied, with the results being represented as odds ratios (OR) and 95% CI. For multiple models, Nagelkerke’s Pseudo R^2^ is presented as an indicator of explained variability.

Given the longitudinal feature of the study, differences in the odds of psychological distress between 2020 and 2021 were estimated through simple logistic regression models using all data available. As each participant contributed with one (2020 or 2021) or two (2020 and 2021) observations for longitudinal analyses, each of them was treated as an individual cluster to account for the non-independence between observations.

Statistical analysis was performed using R software version 4.1.3 [38] and the package survey [39], adopting a level of significance of 5%.

## 3. Results

### 3.1. Sociodemographic and Occupational Characteristics

The sociodemographic and occupational characterization of the participants are shown in Table 1. A total of 2027 completed HCP surveys in T0 and 1843 in T1 were considered valid for this study. Longitudinal follow-up was possible for 598 participants. The median age of the sample in 2020 was 43 years (interquartile range (IQR) = 35–52), while in 2021 it was 44 years (IQR = 37–53). In both periods, the majority were women (T0, 83.4%; T1, 82.5%). Almost all reported having higher education (T0, 94.4%; T1, 94.6%). These participants resided all over the national territory, although the most represented region was the North (T0, 35.4%; T1, 38.8%).

Regarding occupational features, the most represented professional category was nurses (T0, 39.4%; T1, 40.9%). Physicians (T0, 26.0%; T1, 22.5%) and healthcare assistants (T0, 5.7%; T1, 5.8%) together represented almost a third of our sample. Most professionals had patient-facing activity (T0, 83.8%; T1, 85.4%) and around a third of them were frontline workers (T0, 29.3%; T1, 35.0%). Participants mainly worked in the public sector (T0, 89.4%; T1, 92.3%) and about half in a hospital facility (T0, 52.6%; T1, 48.3%). More than one third of the participants in 2020 (T0, 37.4%) and about half of those in 2021 (T1, 49.2%) reported an increased workload (Table 1).

### 3.2. Prevalence of Symptoms of Anxiety, Depression, PTSD and Burnout

Table 2 shows data on the frequency of anxiety, depression, PTSD and burnout symptoms according to sociodemographic and occupational variables.

Globally, the frequency of symptoms of all the selected MH outcomes decreased from 2020 (T0) to 2021 (T1).

A statistically significant decrease of reported symptoms of anxiety was found between T0 and T1 (26.1% to 23.3%, respectively; *p* = 0.028). Regarding T0, anxiety was significantly associated with all considered variables. In T1, with the exceptions of age, income, career and patient-facing activity, all aforementioned variables were still significantly associated with anxiety (Table 2).

With respect to depression, the results did not show a statistically significant decrease of reported symptoms between T0 and T1 (25.3% vs. 23.7%, respectively; *p* = 0.211). Similar to anxiety, depression was significantly associated with all considered variables in T0. In T1, depression symptoms were still significantly associated with all of the aforementioned variables with the exceptions of age, income, career and patient-facing activity (Table 3).

Symptoms of PTSD were reported by 22.7% of the respondents in T0 and by 19.1% in T1. This decrease was statistically significant (*p* = 0.003). In T0, similar to the reported symptoms of anxiety and depression, all the considered variables apart from age were associated with PTSD. Regarding 2021, except for income, career, patient-facing activity and working position, all remaining variables, including age, were associated with PTSD symptoms (Table 4).

Lastly, regarding symptoms of burnout, the results did not show a statistically significant decrease between T0 and T1 (29.8% vs. 29.5%, respectively; *p* = 0.873). In T0, only sex, income and patient-facing activity variables were not statistically associated with burnout. Regarding T1, and in contrast to T0, the frequency of burnout was significantly higher among women (30.7%, *p* = 0.024). All the other variables were still associated with higher frequencies of symptoms of burnout. Both income and patient-facing activity were not statistically associated with burnout in both years (Table 5).

**Table 2:** Moderate to severe symptoms of anxiety among HCPs during the first two years of COVID-19 pandemic in Portugal.

	Anxiety							
	2020				2021			
	n	% (95% CI)	Model 1 ^a^ OR (95% CI)	Model 2 ^b^ aOR (95% CI)	n	% (95% CI)	Model 1 ^a^ OR (95% CI)	Model 2 ^b^ aOR (95% CI)
**Total**	530	26.1 (24.3–28.1)	—	—	429	23.3 (21.4–25.3) *	—	—
**Sex**								
Male	70	20.8 (16.8–25.5) ^$^	—	—	59	18.3 (14.5–23.0) ^$^	—	—
Female	460	27.2 (25.1–29.4)	**1.42 (1.07–1.89)**	1.33 (0.94–1.89)	370	24.3 (22.2–26.5)	**1.43 (1.06–1.95)**	**1.60 (1.12–2.29)**
**Age groups** (years)								
18–29 y	73	32.6 (26.7–39.0) ^$^	—	—	31	24.8 (18.0–33.2)	—	—
30–39 y	142	24.6 (21.2–28.3)	**0.67 (0.48–0.94)**	0.69 (0.45–1.06)	104	20.6 (17.2–24.3)	0.78 (0.50–1.24)	0.66 (0.39–1.10)
40–49 y	156	26.9 (23.4–30.7)	0.76 (0.54–1.06)	0.93 (0.60–1.44)	144	24.2 (20.9–27.8)	0.97 (0.62–1.51)	0.89 (0.54–1.49)
50–59 y	122	26.8 (22.9–31.0)	0.76 (0.53–1.07)	1.15 (0.71–1.86)	110	25.4 (21.5–29.7)	1.03 (0.65–1.64)	1.21 (0.71–2.07)
60 + y	37	19.6 (14.5–25.9)	**0.50 (0.32–0.79)**	0.93 (0.49–1.78)	40	21.7 (16.3–28.3)	0.84 (0.49–1.44)	1.13 (0.56–2.27)
**Monthly income**								
>EUR 2000	82	22.3 (18.3–26.8) ^$^	—	—	80	23.8 (19.5–28.7)	—	—
EUR 1001–2000	314	25.6 (23.2–28.1)	1.20 (0.91–1.58)	1.08 (0.73–1.61)	266	22.7 (20.4–25.2)	0.94 (0.71–1.25)	1.00 (0.64–1.57)
≤EUR 1000	119	32.1 (27.5–37.0)	**1.65 (1.19–2.29)**	1.45 (0.82–2.58)	66	22.6 (18.1–27.8)	0.93 (0.64–1.36)	1.36 (0.72–2.57)
**Professional career**								
Others	145	24.8 (21.4–28.5)	—	—	121	21.3 (18.1–24.9)	—	—
Physician	131	25.0 (21.4–28.8)	1.01 (0.77–1.33)	0.74 (0.48–1.14)	108	26.0 (22.0–30.5)	1.30 (0.97–1.75)	0.87 (0.54–1.41)
Nurse	212	26.6 (23.7–29.8)	1.10 (0.86–1.41)	0.80 (0.56–1.15)	176	23.4 (20.5–26.5)	1.13 (0.87–1.47)	0.78 (0.54–1.12)
Healthcare Assistant	41	35.3 (27.1–44.5)	**1.66 (1.08–2.54)**	0.71 (0.38–1.32)	24	22.6 (15.6–31.7)	1.08 (0.66–1.78)	0.65 (0.34–1.23)
**Patient-facing activity**								
No	55	19.0 (14.8–23.9) ^$^	—	—	57	22.4 (17.6–27.9)	—	—
Yes	415	27.7 (25.5–30.0)	**1.64 (1.19–2.24)**	**1.53 (1.03–2.27)**	357	23.9 (21.8–26.2)	1.09 (0.79–1.50)	1.10 (0.72–1.66)
**Working position**								
Non-frontline	295	23.2 (20.9–25.6) ^$^	—	—	252	22.4 (20.1–24.9) ^$^	—	—
Frontline	171	32.4 (28.6–36.6)	**1.59 (1.27–1.99)**	1.23 (0.93–1.62)	163	26.9 (23.5–30.6)	**1.27 (1.01–1.60)**	1.16 (0.87–1.55)
**Workload**								
No increase	282	22.5 (20.3–24.9) ^$^	—	—	169	18.1 (15.8–20.7) ^$^	—	—
Increase	240	32.1 (28.8–35.5)	**1.63 (1.33–1.99)**	0.97 (0.75–1.27)	258	28.6 (25.7–31.6)	**1.81 (1.45–2.26)**	1.17 (0.87–1.57)
**Needed to move away from family residency**								
No	257	20.3 (18.1–22.6) ^$^	—	—	249	20.9 (18.7–23.3) ^$^	—	—
Yes	265	36.3 (32.8–39.8)	**2.24 (1.83–2.75)**	**1.73 (1.35–2.23)**	178	27.6 (24.3–31.2)	**1.44 (1.16–1.80)**	0.93 (0.71–1.21)
**Work–life balance**								
No difficulties in work–life balance	73	10.8 (8.6–13.3) ^$^	—	—	55	8.8 (6.8–11.2) ^$^	—	—
Difficulties in work–life balance	455	34.3 (31.8–36.9)	**4.32 (3.30–5.65)**	**3.23 (2.33–4.48)**	373	30.8 (28.3–33.5)	**4.64 (3.43–6.28)**	**3.43 (2.37–4.95)**
**Social/family support**								
Lack of support	85	51.5 (43.9–59.1) ^$^	—	—	89	51.7 (44.2–59.2) ^$^	—	—
Good support	445	23.9 (22.0–25.9)	**0.30 (0.21–0.41)**	**0.46 (0.30–0.70)**	339	20.3 (18.4–22.3)	**0.24 (0.17–0.33)**	**0.37 (0.25–0.54)**
**Hobbies and lifestyle**								
Unable to maintain hobbies and lifestyle	370	36.5 (33.5–39.5) ^$^	—	—	297	34.8 (31.7–38.0) ^$^	—	—
Able to maintain hobbies and lifestyle	160	15.8 (13.7–18.2)	**0.33 (0.26–0.40)**	**0.46 (0.36–0.60)**	132	13.3 (11.4–15.6)	**0.29 (0.23–0.36)**	**0.40 (0.31–0.53)**
**Resilience**								
Medium/low level	456	31.4 (29.1–33.9) ^$^	—	—	378	30.8 (28.2–33.4) ^$^	—	—
High level	74	12.8 (10.3–15.8)	**0.32 (0.25–0.42)**	**0.45 (0.33–0.61)**	51	8.3 (6.4–10.8)	**0.20 (0.15–0.28)**	**0.28 (0.20–0.39)**
**Nagelkerke’s pseudo R^2^**	—	—	—	0.219	—	—	—	0.255

OR, odds ratio; CI, confidence interval. Notes: ^a^ unadjusted OR; ^b^ OR adjusted for all variables. **^$^** Statistically significant variables (*p* < 0.05) in bivariate analyses. Statistically significant differences in the odds of the outcome in 2021 compared to 2020: * *p* < 0.05. Statistically significant categories in the regression models (*p* < 0.05) are in bold.

**Table 3:** Moderate to severe symptoms of depression among HCPs during the first two years of COVID-19 pandemic in Portugal.

	Depression							
	2020				2021			
	n	% (95% CI)	Model 1 ^a^ OR (95% CI)	Model 2 ^b^ aOR (95% CI)	n	% (95% CI)	Model 1 ^a^ OR (95% CI)	Model 2 ^b^ aOR (95% CI)
**Total**	513	25.3 (23.5–27.2)	—	—	437	23.7 (21.8–25.7)	—	—
**Sex**								
Male	60	17.9 (14.1–22.3) ^$^	—	—	55	17.1 (13.3–21.6) ^$^	—	—
Female	453	26.8 (24.7–29.0)	**1.68 (1.25–2.27)**	**1.68 (1.16–2.44)**	382	25.1 (23.0–27.4)	**1.63 (1.19–2.23)**	**1.86 (1.28–2.70)**
**Age groups** (years)								
18–29 y	72	32.1 (26.3–38.6) ^$^	—	—	35	28.0 (20.8–36.6)	—	—
30–39 y	140	24.2 (20.9–27.9)	**0.67 (0.48–0.95)**	0.74 (0.48–1.14)	114	22.5 (19.1–26.4)	0.75 (0.48–1.16)	**0.55 (0.33–0.93)**
40–49 y	156	26.9 (23.4–30.7)	0.78 (0.56–1.09)	0.86 (0.55–1.34)	140	23.5 (20.3–27.1)	0.79 (0.51–1.22)	0.65 (0.39–1.10)
50–59 y	112	24.6 (20.8–28.7)	**0.69 (0.48–0.98)**	0.97 (0.60–1.57)	112	25.9 (21.9–30.2)	0.90 (0.57–1.40)	1.00 (0.58–1.72)
60 + y	33	17.5 (12.7–23.6)	**0.45 (0.28–0.71)**	0.89 (0.46–1.72)	36	19.6 (14.4–26.0)	0.63 (0.37–1.07)	0.76 (0.37–1.55)
**Monthly income**								
>EUR 2000	80	21.7 (17.8–26.3) ^$^	—	—	81	24.1 (19.8–29.0)	—	—
EUR 1001–2000	294	24.0 (21.7–26.4)	1.13 (0.86–1.50)	0.86 (0.57–1.29)	270	23.0 (20.7–25.5)	0.94 (0.71–1.25)	0.84 (0.53–1.34)
≤EUR 1000	125	33.7 (29.0–38.7)	**1.83 (1.32–2.54)**	1.09 (0.61–1.93)	73	25.0 (20.3–30.3)	1.05 (0.73–1.51)	1.19 (0.63–2.25)
**Professional career**								
Others	133	22.7 (19.5–26.3) ^$^	—	—	123	21.7 (18.5–25.2)	—	—
Physician	124	23.6 (20.2–27.5)	1.05 (0.79–1.39)	0.72 (0.46–1.11)	105	25.3 (21.3–29.7)	1.23 (0.91–1.65)	0.88 (0.54–1.45)
Nurse	207	26.0 (23.1–29.2)	1.19 (0.93–1.53)	0.93 (0.64–1.34)	182	24.2 (21.2–27.4)	1.15 (0.89–1.50)	0.90 (0.63–1.29)
Healthcare Assistant	47	40.5 (31.9–49.8)	**2.31 (1.52–3.52)**	1.18 (0.63–2.19)	27	25.5 (18.0–34.7)	1.24 (0.76–2.00)	0.83 (0.43–1.59)
**Patient-facing activity**								
No	53	18.3 (14.2–23.2) ^$^	—	—	58	22.7 (18.0–28.3)	—	—
Yes	392	26.2 (24.0–28.5)	**1.58 (1.15–2.18)**	1.32 (0.88–1.97)	357	23.9 (21.8–26.2)	1.07 (0.78–1.47)	0.91 (0.60–1.37)
**Working position**								
Non-frontline	269	21.1 (19.0–23.5) ^$^	—	—	247	22.0 (19.6–24.5) ^$^	—	—
Frontline	178	33.8 (29.9–37.9)	**1.90 (1.52–2.38)**	**1.48 (1.12–1.95)**	167	27.6 (24.1–31.3)	**1.35 (1.08–1.70)**	1.15 (0.86–1.55)
**Workload**								
No increase	257	20.5 (18.4–22.9) ^$^	—	—	165	17.7 (15.3–20.3) ^$^	—	—
Increase	247	33.0 (29.7–36.5)	**1.91 (1.55–2.34)**	1.16 (0.89–1.52)	271	30.0 (27.1–33.1)	**2.00 (1.60–2.49)**	1.17 (0.87–1.58)
**Needed to move away from family residency**								
No	249	19.6 (17.5–21.9) ^$^	—	—	234	19.6 (17.5–22.0) ^$^	—	—
Yes	255	34.9 (31.5–38.4)	**2.19 (1.79–2.70)**	**1.58 (1.22–2.05)**	202	31.3 (27.8–35.0)	**1.87 (1.50–2.33)**	1.25 (0.95–1.64)
**Work–life balance**								
No difficulties in work–life balance	65	9.6 (7.6–12.0) ^$^	—	—	48	7.6 (5.8–10.0) ^$^	—	—
Difficulties in work–life balance	445	33.5 (31.0–36.1)	**4.75 (3.59–6.29)**	**2.96 (2.10–4.17)**	388	32.1 (29.5–34.8)	**5.70 (4.15–7.84)**	**3.90 (2.63–5.78)**
**Social/family support**								
Lack of support	86	52.1 (44.5–59.7) ^$^	—	—	95	55.2 (47.7–62.5) ^$^	—	—
Good support	426	22.9 (21.0–24.9)	**0.27 (0.20–0.38)**	**0.48 (0.31–0.73)**	342	20.5 (18.6–22.5)	**0.21 (0.15–0.29)**	**0.29 (0.20–0.42)**
**Hobbies and lifestyle**								
Unable to maintain hobbies and lifestyle	381	37.5 (34.6–40.6) ^$^	—	—	315	36.9 (33.7–40.2) ^$^	—	—
Able to maintain hobbies and lifestyle	132	13.0 (11.1–15.3)	**0.25 (0.20–0.31)**	**0.36 (0.27–0.47)**	122	12.3 (10.4–14.5)	**0.24 (0.19–0.30)**	**0.35 (0.27–0.47)**
**Resilience**								
Medium/low level	445	30.7 (28.3–33.1) ^$^	—	—	385	31.3 (28.8–34.0) ^$^	—	—
High level	68	11.8 (9.4–14.7)	**0.30 (0.23–0.40)**	**0.45 (0.33–0.61)**	52	8.5 (6.5–11.0)	**0.20 (0.15–0.28)**	**0.29 (0.21–0.41)**
**Nagelkerke’s pseudo R^2^**	—	—	—	0.247	—	—	—	0.299

OR, odds ratio; CI, confidence interval. Notes: ^a^ unadjusted OR; ^b^ OR adjusted for all variables. **^$^** Statistically significant variables (*p* < 0.05) in bivariate analyses. Statistically significant differences in the odds of the outcome in 2021 compared to 2020. Statistically significant categories in the regression models (*p* < 0.05) are in bold.

**Table 4:** Symptoms of post-traumatic stress disorder (PTSD) among HCPs during the first two years of COVID-19 pandemic in Portugal.

	PTSD							
	2020				2021			
	n	% (95% CI)	Model 1 ^a^ OR (95% CI)	Model 2 ^b^ aOR (95% CI)	n	% (95% CI)	Model 1 ^a^ OR (95% CI)	Model 2 ^b^ aOR (95% CI)
**Total**	461	22.7 (21.0–24.6)	—	—	352	19.1 (17.4–21.0) **	—	—
**Sex**								
Male	54	16.1 (12.5–20.4) ^$^	—	—	37	11.5 (8.4–15.5) ^$^	—	—
Female	407	24.1 (22.1–26.2)	**1.66 (1.21–2.26)**	**1.61 (1.12–2.33)**	315	20.7 (18.7–22.8)	**2.01 (1.40–2.90)**	**2.23 (1.48–3.37)**
**Age groups** (years)								
18–29 y	47	21.0 (16.1–26.8)	—	—	25	20.0 (13.8–28.0) ^$^	—	—
30–39 y	130	22.5 (19.3–26.1)	1.09 (0.75–1.59)	1.23 (0.78–1.93)	83	16.4 (13.4–19.9)	0.78 (0.48–1.29)	0.62 (0.36–1.08)
40–49 y	141	24.3 (21.0 -28.0)	1.21 (0.83–1.76)	1.24 (0.78–1.98)	109	18.3 (15.4–21.6)	0.90 (0.55–1.46)	0.72 (0.41–1.24)
50–59 y	114	25.0 (21.2–29.2)	1.26 (0.85–1.85)	**1.87 (1.13–3.08)**	105	24.2 (20.4–28.5)	1.28 (0.78–2.09)	1.45 (0.82–2.57)
60 + y	29	15.3 (10.9–21.3)	0.68 (0.41–1.14)	1.35 (0.69–2.67)	30	16.3 (11.6–22.4)	0.78 (0.43–1.40)	1.26 (0.63–2.53)
**Monthly income**								
>EUR 2000	67	18.2 (14.6–22.5) ^$^	—	—	53	15.8 (12.2–20.1)	—	—
EUR 1001–2000	283	23.1 (20.8–25.5)	**1.35 (1.00–1.81)**	0.97 (0.65–1.46)	224	19.1 (17.0–21.5)	1.26 (0.91–1.75)	0.90 (0.57–1.43)
≤EUR 1000	100	27.0 (22.7–31.7)	**1.66 (1.17–2.35)**	0.97 (0.54–1.75)	64	21.9 (17.5–27.1)	**1.50 (1.00–2.25)**	1.40 (0.72–2.73)
**Professional career**								
Others	126	21.5 (18.4–25.1) ^$^	—	—	108	19.0 (16.0–22.5)	—	—
Physician	98	18.7 (15.6–22.2)	0.84 (0.62–1.12)	**0.55 (0.35–0.85)**	65	15.7 (12.5–19.5)	0.79 (0.56–1.11)	**0.47 (0.28–0.79)**
Nurse	198	24.9 (22.0–28.0)	1.21 (0.94–1.56)	0.92 (0.64–1.32)	154	20.5 (17.7–23.5)	1.10 (0.83–1.44)	0.77 (0.53–1.12)
Healthcare Assistant	38	32.8 (24.8–41.9)	**1.77 (1.15–2.74)**	0.92 (0.47–1.79)	25	23.6 (16.4–32.7)	1.31 (0.80–2.16)	0.78 (0.39–1.54)
**Patient-facing activity**								
No	50	17.2 (13.3–22.1) ^$^	—	—	51	20.0 (15.5–25.4)	—	—
Yes	353	23.6 (21.5–25.8)	**1.48 (1.07–2.05)**	1.16 (0.78–1.73)	287	19.2 (17.3–21.3)	0.95 (0.68–1.33)	0.91 (0.59–1.40)
**Working position**								
Non-frontline	240	18.9 (16.8–21.1) ^$^	—	—	207	18.4 (16.2–20.8)	—	—
Frontline	162	30.7 (26.9–34.8)	**1.91 (1.51–2.41)**	**1.67 (1.26–2.21)**	132	21.8 (18.7–25.3)	1.24 (0.97–1.58)	1.26 (0.92–1.72)
**Workload**								
No increase	253	20.2 (18.1–22.5) ^$^	—	—	141	15.1 (12.9–17.5) ^$^	—	—
Increase	200	26.7 (23.7–30.0)	**1.44 (1.16–1.78)**	0.97 (0.74–1.26)	209	23.1 (20.5–26.0)	**1.69 (1.34–2.15)**	1.05 (0.77–1.43)
**Needed to move away from family residency**								
No	227	17.9 (15.9–20.1) ^$^	—	—	178	14.9 (13.0–17.1) ^$^	—	—
Yes	226	30.9 (27.7–34.4)	**2.05 (1.66–2.54)**	**1.78 (1.37–2.31)**	172	26.7 (23.4–30.2)	**2.07 (1.64–2.62)**	**1.54 (1.15–2.06)**
**Work–life balance**								
No difficulties in work–life balance	86	12.7 (10.4–15.4) ^$^	—	—	39	6.2 (4.6–8.4) ^$^	—	—
Difficulties in work–life balance	374	28.2 (25.8–30.6)	**2.70 (2.09–3.49)**	**1.94 (1.41–2.67)**	311	25.7 (23.3–28.2)	**5.22 (3.69–7.41)**	**3.78 (2.52–5.66)**
**Social/family support**								
Lack of support	81	49.1 (41.5–56.7) ^$^	—	—	71	41.3 (34.1–48.8) ^$^	—	—
Good support	380	20.4 (18.6–22.3)	**0.27 (0.19–0.37)**	**0.37 (0.24–0.55)**	281	16.8 (15.1–18.7)	**0.29 (0.21–0.40)**	**0.44 (0.29–0.67)**
**Hobbies and lifestyle**								
Unable to maintain hobbies and lifestyle	304	30.0 (27.2–32.8) ^$^	—	—	248	29.0 (26.1–32.2) ^$^	—	—
Able to maintain hobbies and lifestyle	157	15.5 (13.4–17.9)	**0.43 (0.35–0.53)**	**0.58 (0.44–0.76)**	104	10.5 (8.7–12.6)	**0.29 (0.22–0.37)**	**0.40 (0.30–0.54)**
**Resilience**								
Medium/low level	394	27.2 (24.9–29.5) ^$^	—	—	310	25.2 (22.9–27.7) ^$^	—	—
High level	67	11.6 (9.3–14.5)	**0.35 (0.27–0.47)**	**0.40 (0.29–0.55)**	42	6.8 (5.1–9.1)	**0.22 (0.16–0.31)**	**0.30 (0.21–0.43)**
**Nagelkerke’s pseudo R^2^**	—	—	—	0.184	—	—	—	0.262

OR, odds ratio; CI, confidence interval. Notes: ^a^ unadjusted OR; ^b^ OR adjusted for all variables. **^$^** Statistically significant variables (*p* < 0.05) in bivariate analyses. Statistically significant differences in the odds of the outcome in 2021 compared to 2020: ** *p* < 0.01. Statistically significant categories in the regression models (*p* < 0.05) are in bold.

**Table 5:** Symptoms of burnout among HCPs during the first two years of COVID-19 pandemic in Portugal.

	Burnout							
	2020				2021			
	n	% (95% CI)	Model 1 ^a^ OR (95% CI)	Model 2 ^b^ aOR (95% CI)	n	% (95% CI)	Model 1 ^a^ OR (95% CI)	Model 2 ^b^ aOR (95% CI)
**Total**	570	29.8 (27.8–31.9)	—	—	543	29.5 (27.5- 31.7)	—	—
**Sex**								
Male	88	27.0 (22.4–32.1)	—	—	78	24.3 (19.9–29.3) ^$^	—	—
Female	482	30.3 (28.1–32.6)	1.18 (0.90–1.54)	1.08 (0.80–1.47)	465	30.7 (28.4–33.0)	**1.38 (1.04–1.82)**	1.35 (0.98–1.86)
**Age groups** (years)								
18–29 y	79	38.2 (31.8–45.0) ^$^	—	—	39	31.2 (23.6–39.9) ^$^	—	—
30–39 y	170	31.3 (27.5–35.3)	0.74 (0.53–1.03)	**0.67 (0.45–0.99)**	148	29.3 (25.5–33.4)	0.91 (0.60–1.40)	0.81 (0.51–1.28)
40–49 y	164	30.5 (26.7–34.5)	**0.71 (0.51–0.99)**	0.78 (0.52–1.17)	186	31.5 (27.8–35.3)	1.01 (0.67–1.54)	1.19 (0.74–1.92)
50–59 y	122	27.4 (23.5–31.8)	**0.61 (0.43–0.87)**	0.72 (0.46–1.13)	134	30.9 (26.8–35.5)	0.99 (0.64–1.52)	1.36 (0.82–2.25)
60 + y	35	19.3 (14.2–25.8)	**0.39 (0.24–0.62)**	**0.53 (0.29–0.99)**	36	19.6 (14.4–26.0)	**0.54 (0.32–0.91)**	0.69 (0.36–1.32)
**Monthly income**								
>EUR 2000	103	28.7 (24.2–33.6)	—	—	100	29.9 (25.2–35.0)	—	—
EUR 1001–2000	349	29.6 (27.1–32.3)	1.05 (0.81–1.36)	0.96 (0.66–1.39)	346	29.6 (27.1–32.3)	0.99 (0.76–1.29)	1.13 (0.73–1.74)
≤EUR 1000	105	32.7 (27.8–38.1)	1.21 (0.87–1.68)	1.01 (0.60–1.71)	84	28.8 (23.8–34.2)	0.95 (0.67–1.34)	1.62 (0.87–3.00)
**Professional career**								
Others	114	23.5 (19.9–27.4) ^$^	—	—	151	26.6 (23.1–30.4) ^$^	—	—
Physician	173	33.3 (29.4–37.5)	**1.63 (1.24–2.16)**	1.25 (0.83–1.88)	145	35.1 (30.6–39.9)	**1.49 (1.13–1.97)**	1.36 (0.86–2.16)
Nurse	241	30.3 (27.2–33.6)	**1.42 (1.10–1.84)**	1.17 (0.82–1.67)	221	29.5 (26.3–32.8)	1.15 (0.90–1.47)	0.80 (0.58–1.10)
Healthcare Assistant	42	36.5 (28.2–45.8)	**1.88 (1.22–2.90)**	1.22 (0.67–2.22)	26	24.5 (17.2–33.7)	0.90 (0.56–1.45)	**0.41 (0.21–0.82)**
**Patient-facing activity**								
No	74	25.5 (20.8–30.9)	—	—	67	26.3 (21.2–32.1)	—	—
Yes	462	30.8 (28.6–33.2)	1.30 (0.98–1.73)	1.08 (0.76–1.53)	453	30.4 (28.1–32.7)	1.22 (0.91–1.65)	1.13 (0.77–1.67)
**Working position**								
Non-frontline	327	25.7 (23.4–28.2) ^$^	—	—	315	28.0 (25.4–30.7) ^$^	—	—
Frontline	205	38.9 (34.8–43.1)	**1.84 (1.48–2.29)**	**1.40 (1.08–1.82)**	205	33.8 (30.2–37.7)	**1.31 (1.06–1.63)**	1.03 (0.78–1.36)
**Workload**								
No increase	309	25.9 (23.5–28.5) ^$^	—	—	218	23.3 (20.7–26.2) ^$^	—	—
Increase	261	36.0 (32.6–39.6)	**1.61 (1.32–1.96)**	1.02 (0.79–1.32)	324	35.9 (32.8–39.1)	**1.84 (1.50–2.25)**	1.07 (0.82–1.41)
**Needed to move away from family residency**								
No	288	24.0 (21.6–26.5) ^$^	—	—	292	24.5 (22.1–27.0) ^$^	—	—
Yes	282	39.5 (36.0–43.1)	**2.07 (1.69–2.53)**	**1.50 (1.18–1.91)**	250	38.8 (35.1–42.6)	**1.95 (1.59–2.40)**	**1.38 (1.07–1.78)**
**Work–life balance**								
No difficulties in work–life balance	93	14.8 (12.2–17.8) ^$^	—	—	75	12.0 (9.6–14.8) ^$^	—	—
Difficulties in work–life balance	473	37.3 (34.7–40.0)	**3.44 (2.68–4.41)**	**2.10 (1.56–2.81)**	466	38.6 (35.9–41.4)	**4.63 (3.55–6.06)**	**3.38 (2.45–4.66)**
**Social/family support**								
Lack of support	88	59.1 (50.9–66.7) ^$^	—	—	98	57.0 (49.4–64.2) ^$^	—	—
Good support	481	27.3 (25.2–29.4)	**0.26 (0.18–0.37)**	**0.37 (0.25–0.56)**	445	26.7 (24.7–28.9)	**0.28 (0.20–0.38)**	**0.42 (0.29–0.61)**
**Hobbies and lifestyle**								
Unable to maintain hobbies and lifestyle	410	42.0 (38.9–45.1) ^$^	—	—	358	42.2 (38.9–45.5) ^$^	—	—
Able to maintain hobbies and lifestyle	160	17.0 (14.8–19.6)	**0.28 (0.23–0.35)**	**0.41 (0.32–0.52)**	185	18.7 (16.4–21.3)	**0.32 (0.26–0.39)**	**0.47 (0.37–0.60)**
**Resilience**								
Medium/low level	499	36.3 (33.8–38.8) ^$^	—	—	470	38.4 (35.7–41.2) ^$^	—	—
High level	71	13.2 (10.6–16.3)	**0.27 (0.20–0.35)**	**0.35 (0.26–0.48)**	73	11.9 (9.6–14.7)	**0.22 (0.17–0.28)**	**0.30 (0.22–0.40)**
**Nagelkerke’s pseudo R^2^**	—	—	—	0.234	—	—	—	0.269

OR, odds ratio; CI, confidence interval. Notes: ^a^ unadjusted OR; ^b^ OR adjusted for all variables. **^$^** Statistically significant variables (*p* < 0.05) in bivariate analyses. Statistically significant differences in the odds of the outcome in 2021 compared to 2020. Statistically significant categories in the regression models (*p* < 0.05) are in bold.

### 3.3. Risk and Protective Mental Health Factors

Table 2, Table 3, Table 4 and Table 5 present the OR and 95% CI for the likelihood of experiencing anxiety, depression, PTSD and burnout symptoms, in relation to sociodemographic and occupational variables, COVID-19-related experiences, protective behaviors and resilience in each year (unadjusted OR: model 1; adjusted OR (aOR) for all variables in the model: model 2).

Simple logistic regression models showed that all the selected variables except for professional career were associated with experiencing symptoms of anxiety in T0. In T1, being a female, working at the frontline, increased workload, the need to move away from family residency and having work–life imbalance were significantly associated with an increased likelihood of experiencing symptoms of anxiety (Table 2). In the multiple adjusted model (model 2; Table 2) the variables of being patient-facing (aOR, 1.53; 95% CI, 1.03–2.27), the need to move away from family residency (aOR, 1.73; 95% CI, 1.35–2.23) and work–life imbalance (aOR, 3.23; 95% CI, 2.33–4.48) were significantly associated with symptoms of anxiety in T0. Moreover, in T1, only being female (aOR, 1.60; 95% CI, 1.12–2.29) and the perception of work–life imbalance (aOR, 3.43; 95% CI, 2.37–4.95) were negatively associated with experiencing symptoms of anxiety.

Concerning symptoms of depression, simple logistic regression models (unadjusted OR, model 1; Table 3) indicated that all considered variables were associated with a negative outcome in 2020. In 2021, being a female, at the frontline, increased workload, the need to move away from family residency and work–life imbalance were found to be significantly associated with an increased likelihood of experiencing symptoms of anxiety (Table 3). Moreover, in the adjusted model (model 2), being a female was associated with increased symptoms of depression both in T0 and T1 (aOR, 1.68; 95% CI, 1.16–2.44; aOR, 1.86; 95% CI, 1.28–2.70, respectively), as well as work–life imbalance (aOR, 2.96; 95% CI, 2.10–4.17; aOR, 3.90; 95% CI, 2.63–5.78, T0 and T1, respectively). The results also indicate that working at the frontline (aOR, 1.48; 95% CI, 1.12–1.95) and moving away from family residency (aOR, 1.58; 95% CI, 1.22–2.05) were associated with symptoms of depression in T0. Considering T1, 30–39-year-old respondents had a lower likelihood of depression compared to those aged 18–29 (aOR, 0.55; 95% CI, 0.33–0.93).

In terms of symptoms of PTSD, similarly to the previous outcomes investigated, simple logistic regression models (unadjusted OR, model 1; Table 4) revealed that, except for age, all selected variables were associated with PTSD in T0. Regarding T1, being a female, having a monthly income of ≤EUR 1000 (compared to those with an income >EUR 2000 /month), increased workload, moving away from family residency and work–life imbalance were significantly associated with an increased likelihood of experiencing symptoms of PTSD (Table 4). In the adjusted multiple models (model 2), being a female (aOR, 1.61; 95% CI, 1.12–2.33; aOR, 2.23; 95% CI, 1.48–3.37, T0 and T1, respectively), moving away from family residency (aOR, 1.78; 95% CI, 1.37–2.31; aOR, 1.54; 95% CI, 1.15–2.06, T0 and T1, respectively) and work–life imbalance (aOR, 1.94; 95% CI, 1.41–2.67; aOR, 3.78; 95% CI, 2.52–5.66, T0 and T1, respectively) were significantly associated with an increased likelihood of experiencing symptoms of PTSD in both years. Furthermore, working at the frontline was only significant in T0 (aOR, 1.67; 95% CI, 1.26–2.21).

When testing for the association between each variable independently and experiencing symptoms of burnout, simple logistic regression models (unadjusted OR, model 1; Table 5) showed that age, career, working position, workload, need to move away from family residency and work–life imbalance were significantly associated with burnout in both years. Furthermore, being a female increased the likelihood of burnout in 2021. In the multiple adjusted model (model 2), moving away from family residency (aOR, 1.50; 95% CI, 1.18–1.91; aOR, 1.38; 95% CI, 1.07–1.78, T0 and T1, respectively) and work–life imbalance (aOR, 2.10; 95% CI, 1.56–2.81; aOR, 3.38; 95% CI, 2.45–4.66, T0 and T1, respectively) were significantly associated with symptoms of burnout in both years. Compared to younger professionals, those aged 30–39 (aOR, 0.67; 95% CI, 0.45–0.99) and ≥60 (aOR, 0.53; 95% CI, 0.29–0.99), as well as frontline HCPs (aOR, 1.40; 95% CI, 1.08–1.82), were only associated with experiencing symptoms of burnout in T0, while professional career was only significant in T1.

When addressing protective MH factors, perceiving good social/family support, being able to maintain hobbies and lifestyle and having a high resilience level were consistently associated with lower likelihood of the symptoms of anxiety, depression, PTSD and burnout in both years, and regardless of the regression models adopted

## 4. Discussion

The present study aimed to characterize Portuguese HCP mental health over two years, estimating the percentage of symptoms of anxiety, depression, PTSD and burnout, and identifying related risk and protective factors. To the best of our knowledge, this is the first study addressing a comprehensive examination of risk and protective MH factors of HCPs beyond sociodemographic and occupational characteristics with a longitudinal component in Portugal.

The study was carried out as a web-based survey, disseminated via institutional email. This approach is subject to potential selection bias, which may have been mitigated by the large number of respondents, in both moments. The study employed self-administered MH screening tools as appropriate for studying large samples. These tools do not substitute a direct psychiatric assessment or provide a clinical diagnosis, which was not the aim of the study, but report useful indicators of MH in a large sample of HCPs. Despite the extensive number of factors examined and their association with MH, other factors could also play an important role in protecting or deteriorating HCP MH during the pandemic, such as social media exposure and social network interactions.

A significant proportion of participants reported moderate-to-severe symptoms of anxiety, depression, PTSD and burnout in the first two years of the COVID-19 pandemic in Portugal. However, these frequencies decreased from 2020 to 2021. The results indicate that in an early stage of the pandemic, high levels of distress may have been an adaptive defense mechanism response to potentially threatening and uncertain events, as well as reflecting changes in HCPs’ professional and personal routines [7,40].

Furthermore, our results revealed several sociodemographic and occupational variables and a set of modifiable common factors that were significantly associated with mental health outcomes in Portuguese HCPs. Being a woman, working on a frontline position and perceived work–life imbalance increased the risk of HCP distress in both T0 and T1.

For all MH outcomes, in both years, with the exception of burnout in T0, the percentage of moderate-to-severe symptoms was higher among women. Women had an increased likelihood of experiencing symptoms of distress than men. Our findings are consistent with evidence elsewhere, consistently reporting a higher prevalence and severity of distress in women [10,12,15,41,42]. Because of the double roles of women in the family organization and professional performance, women may have struggled more when performing their work during the COVID-19 period than their male colleagues may. This gender-related HCP distress should be considered as a psychosocial warning message [41]. Additionally, these outcomes call for the implementation of gender-specific actions to prevent and address MH risk factors.

Overall, frontline HCPs showed worse mental health during both moments. Frontline workers had an increased likelihood of anxiety, depression, PTSD and burnout than non-frontline HCPs. Due to the uncertainty of pandemic evolution and impact, fear of direct contact with virus and infection, and hardship caused by difficult clinical decisions and patient loss, being a frontline worker was a risk factor for psychological distress. This finding is also aligned with other studies [15,16,17,43,44]. For this reason, more attention should be given to frontline HCPs during public health crises. Timely screening and tailored interventions are crucial to prevent mental health burden [45], and policies need to be developed to address these needs.

It is worth noting that HCPs experienced a high level of psychological distress during the two-year period of COVID-19 surveyed, suggesting that high personal and emotional involvement facing this period was felt by all HCPs due to an increased exposure to occupational stressors during the pandemic.

A closer look at the association between MH outcomes and professional careers showed statistically significant differences for depression and PTSD at T0, and for burnout in both T0 and T1, in our sample. In more detail, and focusing on logistic regression models, in T0, healthcare assistants were more likely to have a negative outcome (anxiety, depression and PTSD) than professionals in other categories, whilst physicians were less likely to have a negative outcome (PTSD, T0 and T1) than others. However, physicians, nurses and healthcare assistants were more likely to have high levels of burnout than other HCPs. In T1, the likelihood of physicians having a negative outcome was higher than other HCPs, whereas healthcare assistants were less likely to have a negative outcome than other professionals. These results might indicate that in distinct moments of the pandemic progression, different professionals were at higher risk of distress due to their professional roles and duties. Therefore, tailored training and simulation exercises should be provided to HCPs, according to their career and roles, to improve their understanding and perceptions of MH symptoms and how to seek support. Excessive workload was broadly associated with psychological symptoms, linked with anxiety, depression, PTSD and burnout symptoms in 2020 and 2021. For this reason, ensuring appropriate working hours, reasonable rest periods and rotating shifts for workers are of crucial importance.

MH impairments found among HCPs were also associated with other work-related factors. Perceived work–life imbalance was strongly associated with all the assessed outcomes. Recent research by Voorspoels et al. [46] in Belgium estimated that around 4/10 HCPs experienced psychological distress during the pandemic, and that this scenario might have been avoided if all work-related risk factors were controlled, with individual risk reduced by a factor close to 4. In our study, perceived work–life balance disruptions may be the most intriguing MH risk factor for HCP distress. This result highlights the need for organizational actions towards work–life imbalance mitigation due to its strong effect on MH burden.

The frequency of burnout complaints among HCPs were found to be high, but with no significant differences between the two moments. All HCPs were likely to experience increased exposure to workplace stressors (such as higher workloads and a perceived work–life imbalance) during the first two years of the pandemic. Our results are consistent with other longitudinal research targeting symptoms of burnout in HCPs [40]. These findings stress that the high rates of burnout might persist or even increase in HCPs. Given the impact of these symptoms upon professional performance, urgent interventions to address and mitigate burnout are required for all HCPs.

Protective MH factors were also identified in this study. Higher resilience level and the capacity to maintain hobbies and lifestyle were significantly associated with lower odds of all the MH outcomes in both years.

A protective effect of perceived good social/family support against the development or presence of distress symptoms was also found. Our results demonstrate that social/family support was associated with lower occurrence of symptoms of anxiety, depression, PTSD and burnout in both years. In line with previous studies, social support was consistently found to be a protective MH factor during the COVID-19 pandemic [8,21,46,47,48,49] and in previous viral outbreaks [43,44]. Moreover, individuals with higher levels of social support may feel that they could get the help they needed when facing stressful events. This perception would enhance their beliefs that they would be able to deal with COVID-19 hurdles, which further leads to higher levels of resilience [48].

Resilience was shown to play a mediating role for HCPs facing the pandemic [48]. Previous research found that higher resilience scores were associated with a lower likelihood of generalized anxiety and depression during the COVID-19 pandemic [9,15,22]. Furthermore, our results indicated that higher levels of resilience decreased the likelihood of severe-to-moderate symptoms of anxiety and depression, as well as symptoms of PTSD and burnout, in both years. Improving resilience through training may enhance adaptive coping responses against distress by fostering subjective wellbeing [50].

## 5. Conclusions

The longitudinal approach of our study allowed the following-up of changes in the MH of Portuguese HCPs, during the first two years of the COVID-19 pandemic in Portugal, providing sound evidence to support recommendations addressing this important issue. Although the percentage of moderate-to-severe symptoms of anxiety and depression, PTSD and burnout decreased from 2020 to 2021, our results show that the MH toll of the pandemic in Portuguese HCPs is still high after two years.

Beyond psychological distress, we collected information on a number of under-examined factors, such as individual COVID-19 experiences, lifestyle and social support, and explored their association with MH outcomes. Overall, a set of fixed and modifiable factors were found to increase the odds of distress during the pandemic crisis period: namely, being a woman, working in a frontline position and perception of work–life imbalance. On the other hand, a high resilience level and good social/family support were found to have a protective effect against distress symptoms in our sample.

These findings are crucial to understand the psychological burden of HCPs in Portugal and advocate the need of tailored interventions targeting gender and each professional group during the pandemic.

Protecting the mental health of HCPs is key for safeguarding the provision of sustainable healthcare services, especially during pandemic outbreaks. In the future, further studies to understand the potential long-lasting psychological burden related to COVID-19 among HCPs are needed. The reproduction of similar studies in other countries and cultures, and the combination of data from peri- and post-pandemic crises are encouraged.

Overall, the present results show that performing as a HCP during the pandemic may result in long-term effects on MH. Attenuating these risks may protect health professionals, reducing the possibility of long-lasting psychiatric morbidity.

## Figures and Tables

**Table 1:** Sociodemographic and occupational characterization of participants.

	2020	2021
	n (%)	n (%)
**Overall**	2027 (100%)	1843 (100%)
**Sociodemographic**		
**Sex**		
Male	336 (16.6%)	322 (17.5%)
Female	1691 (83.4%)	1521 (82.5%)
**Age group** (years)		
18–29	224 (11.1%)	125 (6.8%)
30–39	578 (28.5%)	506 (27.5%)
40–49	580 (28.6%)	595 (32.3%)
50–59	456 (22.5%)	433 (23.5%)
+60	189 (9.3%)	184 (10.0%)
**Region ^a^**		
North	718 (35.4%)	715 (38.8%)
Center	462 (22.8%)	384 (20.8%)
Lisbon Metropolitan Area	668 (33.0%)	479 (26.0%)
Alentejo	90 (4.4%)	50 (2.7%)
Algarve	45 (2.2%)	43 (2.3%)
Azores	38 (1.9%)	28 (1.5%)
Madeira	6 (0.3%)	144 (7.8%)
**Education level**		
Higher education	1914 (94.4%)	1744 (94.6%)
Basic/upper secondary education	113 (5.6%)	99 (5.4%)
**Monthly income**		
≤EUR 1000	371 (18.9%)	292 (16.2%)
EUR 1001–2000	1227 (62.4%)	1172 (65.1%)
>EUR 2000	368 (18.7%)	336 (18.7%)
**Lives accompanied**		
No	252 (12.4%)	205 (11.1%)
Yes	1775 (87.6%)	1638 (88.9%)
**Occupational**		
**Professional career**		
Physician	525 (26.0%)	415 (22.5%)
Nurse	796 (39.4%)	753 (40.9%)
Healthcare Assistant	116 (5.7%)	106 (5.8%)
Other ^1^	585 (28.9%)	568 (30.8%)
**Sector ^b^**		
Public	1732 (89.4%)	1652 (92.3%)
Private/Social	206 (10.6%)	138 (7.7%)
**Work at hospital**		
No	960 (47.4%)	953 (51.7%)
Yes	1067 (52.6%)	890 (48.3%)
**Patient-facing activity**		
Yes	1498 (83.8%)	1492 (85.4%)
No ^2^	290 (16.2%)	255 (14.6%)
**Working position**		
Frontline	527 (29.3%)	606 (35.0%)
Non-frontline	1273 (70.7%)	1125 (65.0%)
**Workload**		
Increase	748 (37.4%)	903 (49.2%)
No increase	1252 (62.6%)	934 (50.8%)

Notes: **^a^** Based on the Nomenclature of Territorial Units for Statistics (NUTS) system for Portugal, subdivision II. **^b^** Portugal has a public, universal and general National Health Service (SNS). ^1^ Include hospital administrators, pharmacists, and other health professional careers (e.g., laboratory and diagnostic technicians, other health technicians), etc. ^2^ HCPs not directly in contact with patients.

## Data Availability

The data supporting these findings are available from the corresponding author upon reasonable request. The data are not publicly available due to privacy reasons.

## Supplementary Materials

The following supporting information can be downloaded at: https://www.mdpi.com/article/10.3390/ijerph20043131/s1
