# Supplementary material for: Mental Health of Healthcare Professionals: Two Years of the COVID-19 Pandemic in Portugal
Source: Int J Environ Res Public Health. 2023 Feb 10;20(4):3131. doi: 10.3390/ijerph20043131 (PMC9968046; doi:10.3390/ijerph20043131)
Supplement: Supplementary file 1 [file ijerph-20-03131-s001.zip › ijerph-2196962-supplementary.pdf]

Saúde Mental em Tempos de Pandemia (SM-COVID19)

A situação de pandemia COVID-19 que se vive de momento em Portugal e no mundo tem exigido a todos nós alterações profundas no nosso modo de vida, com possíveis impactos na saúde mental e bem-estar, muito em particular para as pessoas que estão na primeira linha de combate à pandemia.

Por esta razão, o Instituto Nacional de Saúde Doutor Ricardo Jorge, em colaboração com o Instituto de Saúde Ambiental da Faculdade de Medicina da Universidade de Lisboa e com a Sociedade Portuguesa de Psiquiatria e Saúde Mental, está a realizar um estudo sobre a saúde mental e bem-estar em tempos de pandemia, de forma a contribuir para melhorar as respostas do sistema de saúde.

Se tem 18 ou mais anos de idade, a sua participação é muito importante para nós. Para isso pedimos-lhe que responda a um questionário que demora entre 15 e 20 minutos.

As respostas ao questionário são anónimas e os dados solicitados não permitem a identificação de quem responde. A recolha e armazenamento dos dados cumpre o Regulamento Geral de Proteção de Dados, e estes estarão acessíveis apenas à equipa de investigação. As respostas serão analisadas no conjunto de todos os participantes, e nunca de forma individual.

No final do questionário poderá decidir se permite ou não um eventual contacto posterior, para repetir o preenchimento do mesmo. A necessidade de contacto posterior implica que os dados que venha a fornecer nessa fase sejam codificados, sendo o seu tratamento legalmente admitido, nos termos e para os efeitos do Regulamento Geral de Proteção de Dados, através deste consentimento.

Este estudo teve o parecer positivo da Comissão de Ética para a Saúde do Instituto Nacional de Saúde Doutor Ricardo Jorge a 6 de maio de 2020.

Para responder a este questionário confirme que aceita as condições de participação, clicando primeiro na caixa (☐) que surge em baixo, antes de "Declaro que li e que aceito as condições da minha participação" e clicando depois no botão "Seguinte" no final da página.

Em caso de dúvida, pode contactar a equipa de investigação através do endereço smcovid19.info@insa.min-saude.pt

Em nome de toda a equipa agradeço, desde já, a sua participação!

Teresa Caldas de Almeida
Coordenadora do estudo
Departamento de Promoção da Saúde e Prevenção das Doenças Não-Transmissíveis
Instituto Nacional de Saúde Doutor Ricardo Jorge

• Compreendi os objetivos e o que me é solicitado em termos de participação atendendo à finalidade do questionário

• Sei que posso interromper o preenchimento do questionário sem que os dados que já tenha introduzido fiquem registados

☐ Declaro que li e que aceito as condições da minha participação

LIDAR COM A PANDEMIA

A pandemia tem impacto em cada um de nós. Queremos saber um pouco sobre a sua situação pessoal face à pandemia.

1. Em que **situação se encontra** face à **pandemia**?

○ Não estou nem nunca estive infetado/a com o novo coronavírus (que eu saiba)
○ Estou em quarentena/vigilância ativa das Autoridades de Saúde (por ter tido contacto, direto ou indireto, com casos positivos ou com elevada probabilidade de infeção com o novo coronavírus)*
○ Estou em isolamento/confinamento obrigatório (com diagnóstico confirmado de COVID-19)*
○ Estou recuperado já sem sintomas da COVID-19 mas ainda em confinamento*
○ Estou recuperado já sem sintomas da COVID-19 e sem medidas de confinamento*

[Filtro] *1.1. Que **tipo de intervenção recebeu?**

○ Isolado no domicílio
○ Internamento hospitalar
○ Internamento nos cuidados intensivos

2. Algum dos seus **familiares mais próximos esteve infetado** com o novo coronavírus ou **doente com COVID-19?**

○ Sim, esteve em isolamento domiciliário
○ Sim, foi internado no hospital
○ Sim, foi internado no hospital e esteve nos cuidados intensivos
○ Não

SAÚDE E BEM-ESTAR

3. Até que ponto considera que a **situação atual** em que se encontra **face à pandemia** tem **impacto no seu bem-estar em geral**? (de 0 "Absolutamente nada" a 10 "Muitíssimo")

0 10

4. De uma maneira geral, **como considera o seu estado de saúde**?

O Muito bom

O Bom

O Razoável

O Mau

O Muito mau

5. Tem algum dos seguintes problemas de saúde? *[Clique nos itens que se aplicam ao seu caso]*

O Hipertensão arterial (tensão alta)
O Outra doença cardiovascular (problemas cardíacos, ter sofrido um AVC, ...)
O Diabetes
O Doença respiratória (asma, doença obstrutiva crónica, ...)
O Cancro
O Doença autoimune (lupus, Sjögren, doença de Crohn,...)
O Doença neurológica (esclerose múltipla, doença de Parkinson, ...)
O Obesidade
O Depressão
O Ansiedade crónica
*O Outro problema de saúde mental
**O Outro/s problema/s de saúde
O Não tenho nenhum problema de saúde

[Filtro] *5.1.a. Que **outros problemas de saúde mental?**

____

[Filtro] **5.1.b. Que **outros problemas de saúde?**

____

6. Nas <u>últimas duas semanas</u>...

	Sempre	A maior parte do tempo	Bastante tempo	Algum tempo	Pouco tempo	Nunca
... quanto tempo se sentiu muito nervoso/a?	O	O	O	O	O	O
... quanto tempo se sentiu tão deprimido/a que nada o/a animava?	O	O	O	O	O	O
... quanto tempo se sentiu calmo/a e tranquilo/a?	O	O	O	O	O	O
... quanto tempo se sentiu triste/ desanimado/a e em baixo/abatido/a?	O	O	O	O	O	O
... quanto tempo se sentiu feliz?	O	O	O	O	O	O

(MHI-5. Pais-Ribeiro, 2011)

7. Nas <u>últimas duas semanas</u>, em **quantos dias foi afetado/a** pelos **seguintes problemas**?

	Nunca	Em vários dias*	Em mais de metade do número de dias*	Em quase todos os dias*
Senti-me nervoso/a, ansioso/a ou irritado/a	O	O	O	O
Fui incapaz de parar de me preocupar ou de controlar as preocupações	O	O	O	O
Preocupei-me demais com diferentes assuntos	O	O	O	O
Tive dificuldade em relaxar	O	O	O	O
Estive tão inquieto/a que era difícil ficar sossegado/a	O	O	O	O
Estive facilmente incomodado/a ou irritável	O	O	O	O
Senti receio, como se algo terrível pudesse acontecer	O	O	O	O

(GAD-7; Sousa et al., 2015)

[Filtro] *7.1. **Nestas últimas semanas, começou a tomar, diminuiu ou aumentou a quantidade de medicamentos**, por causa dos **problemas indicados na pergunta anterior**?

O Não
O Sim, comecei a tomar medicamentos (que não tomava antes)
O Diminuí a quantidade de medicação
O Aumentei a quantidade de medicação

8. Durante as <u>**últimas duas semanas**</u>**, em quantos dias foi afetado/a** por **algum dos seguintes problemas**?

	Nunca	Em vários dias*	Em mais de metade do número de dias*	Em quase todos os dias*
Tive pouco interesse ou prazer em fazer coisas	O	O	O	O
Senti desânimo, desalento ou falta de esperança	O	O	O	O
Tive dificuldade em adormecer ou em dormir sem interrupções, ou dormi demais	O	O	O	O
Senti cansaço ou falta de energia	O	O	O	O
Tive falta ou excesso de apetite	O	O	O	O
Senti que não gosto de mim próprio/a — ou que sou um/a falhado/a ou me desiludi a mim próprio/a ou à minha família	O	O	O	O
Tive dificuldade em concentrar-me nas coisas, como ao ler o jornal ou ver televisão	O	O	O	O
Movimentei-me ou falei tão lentamente que outras pessoas poderão ter notado. Ou o oposto: estive agitado/a a ponto de andar de um lado para o outro muito mais do que é habitual	O	O	O	O
Pensei que seria melhor estar morto/a, ou em magoar-me a mim próprio/a de alguma forma	O	O	O	O

(PHQ-9; Monteiro et al., 2013)

[Filtro] *8.1. **Até que ponto** estes **problemas dificultaram** o seu **trabalho, o cuidar da casa** ou o **lidar com outras pessoas**?

Não dificultaram	Dificultaram um pouco	Dificultaram muito	Dificultaram extremamente
O	O	O	O

[Filtro] *8.2. Nestas <u>últimas duas semanas</u>, **começou a tomar, diminuiu ou aumentou a quantidade de medicamentos que tomou**, por causa desses problemas indicados na pergunta anterior?

O Não
O Sim, comecei a tomar medicamentos (que não tomava antes)
O Diminuí a quantidade de medicação
O Aumentei a quantidade de medicação

9. Nas <u>últimas duas semanas</u>, diria que **tem aumentado, mantido ou diminuído** o seu...

	Mais do que o habitual	O mesmo	Menos do que o habitual	Não se aplica
Consumo de café	O	O	O	O
Consumo de doces (chocolate, ...)	O	O	O	O
Consumo de bebidas alcoólicas	O	O	O	O
Consumo de tabaco	O	O	O	O
Consumo de *cannabis* (haxixe, ...)	O	O	O	O
Consumo de outras substâncias	O	O	O	O
Tempo dedicado a videojogos	O	O	O	O
Apostas / jogos a dinheiro	O	O	O	O
Tempo dedicado a pornografia (internet, filmes, ...)	O	O	O	O

10. **Até que ponto concorda** com as seguintes afirmações enquanto **se aplicam à sua realidade e a si** nestas <u>últimas duas semanas</u>. **Se alguma destas situações não ocorreu recentemente, responda de acordo com o que pensa que teria sentido caso tivessem ocorrido.**

	Nada verdadeira	Raramente verdadeira	Às vezes verdadeira	Geralmente verdadeira	Quase sempre verdadeira

Eu sou capaz de me adaptar quando ocorrem mudanças	○	○	○	○	○
Eu consigo lidar com qualquer coisa que aconteça na minha vida	○	○	○	○	○
Eu tento ver as coisas com humor quando me deparo com problemas	○	○	○	○	○
Ter de lidar com o *stress* torna-me mais forte	○	○	○	○	○
Tenho tendência para recuperar rapidamente depois de períodos com doença, ferimentos ou outras dificuldades	○	○	○	○	○
Eu acredito que posso atingir os meus objetivos, mesmo que existam obstáculos	○	○	○	○	○
Sob pressão, mantenho-me focado/a e a pensar com clareza	○	○	○	○	○
Eu não sou facilmente desencorajado/apelo insucesso	○	○	○	○	○
Eu penso em mim como uma pessoa forte ao lidar com os desafios e dificuldades da vida	○	○	○	○	○
Eu sou capaz de lidar com sentimentos desagradáveis ou dolorosos como a	○	○	○	○	○

tristeza, o medo e a raiva

(CD-RISC 10, Almeida et al., 2020)

11. Encontra de seguida uma **lista de problemas** que por vezes as **pessoas têm em resposta a um acontecimento muito stressante**. Por favor, leia atentamente cada problema listado e **indique até que ponto tem sido incomodado/a** por cada um destes problemas nas <u>**últimas duas semanas**</u>.

	Nada	Um pouco	Modera-damente	Bastante	Muito
De repente sentiu-se ou agiu como se o acontecimento stressante estivesse novamente a acontecer ou como se estivesse a vivê-lo outra vez?	○	○	○	○	○
Evitou pessoas, locais, conversas, atividades, objetos ou situações que o/a lembram do acontecimento stressante?	○	○	○	○	○
Sentiu-se distante ou afastado/a das outras pessoas?	○	○	○	○	○
Teve comportamentos de irritabilidade, explosões de fúria ou foi agressivo/a?	○	○	○	○	○

(Zuromski et al. 2019)

ALTERAÇÕES DE VIDA

Devido à pandemia COVID19, muitos dos nossos hábitos e formas de viver tiveram de ser alterados. Tendo em conta a sua experiência, pedimos-lhe que assinale até que ponto concorda com cada uma das afirmações abaixo.

12. **Até que ponto concorda** com cada uma das seguintes **afirmações** sobre **conciliação entre trabalho e família, durante este período de pandemia**:

	Não concordo de todo	Não concordo	Não sei ao certo	Concordo	Concordo totalmente	Não se aplica ao meu caso
Tenho conseguido conciliar o meu trabalho com as tarefas da vida doméstica	○	○	○	○	○	○
Tenho conseguido conciliar o meu trabalho com o acompanhamento dos menores a meu cargo filhos ou outros (até aos 5 anos)	○	○	○	○	○	○
Tenho conseguido conciliar o meu trabalho com o acompanhamento escolar dos menores a meu cargo filhos ou outros (entre os 6 e os 10 anos)	○	○	○	○	○	○
Tenho conseguido conciliar o meu trabalho com o acompanhamento escolar dos menores a meu cargo filhos ou outros (entre os 11 e os 17 anos)	○	○	○	○	○	○
Acontece-me trabalhar mais vezes (do que antes da pandemia) fora do horário normal: aos fins-de-semana, ao serão…	○	○	○	○	○	○

O meu trabalho tem interferido com a minha vida familiar e pessoal (mais do que antes da pandemia)	○	○	○	○	○	○

13. **Até que ponto concorda** com cada uma das seguintes **afirmações** relativas ao seu **trabalho e rendimento financeiro**, nesta **altura de pandemia:**

	Não concordo de todo	Não concordo	Não sei ao certo	Concordo	Concordo totalment e	Não se aplica ao meu caso
Tive de encontrar formas alternativas para manter a minha atividade profissional	○	○	○	○	○	○
Consigo que a minha atividade profissional tenha o mesmo nível de desempenho/produtivi dade	○	○	○	○	○	○
Tenho todas as condições que preciso para desempenhar a minha atividade profissional em segurança	○	○	○	○	○	○
Mantenho o rendimento financeiro que tinha antes da pandemia	○	○	○	○	○	○
Consigo ter a mesma capacidade financeira que tinha antes da pandemia para comprar bens indispensáveis e pagar as contas	○	○	○	○	○	○

14. **Até que ponto concorda** com cada uma das seguintes **afirmações** relativas a **apoio social e familiar**, nesta **altura de pandemia:**

	Não concordo de todo	Não concordo	Não sei ao certo	Concordo	Concordo totalmente	Não se aplica ao meu caso

Tenho acesso fácil a apoio social domiciliário	○	○	○	○	○	○
Desde que começou a pandemia, sinto que tenho o mesmo apoio por parte das pessoas que são importantes para mim	○	○	○	○	○	○
Desde que começou a pandemia, consigo dar o apoio que gostaria de dar a pessoas que são importantes para mim	○	○	○	○	○	○
Tenho tido muita compreensão e apoio por parte das pessoas que são importantes para mim	○	○	○	○	○	○
Tenho pelo menos uma pessoa próxima com quem posso contar sempre que preciso	○	○	○	○	○	○
Quando estou doente sei que posso, sem qualquer hesitação, pedir apoio a familiares ou amigos	○	○	○	○	○	○
Quando estou em baixo, sei a quem recorrer sem qualquer hesitação	○	○	○	○	○	○
Consigo gerir as minhas co-responsabilidades parentais (guarda partilhada, visitas, etc.)	○	○	○	○	○	○
Consigo gerir bem o convívio permanente com as pessoas com quem vivo	○	○	○	○	○	○
Consigo continuar a dar apoio a familiares ou outras pessoas que estão a meu cargo	○	○	○	○	○	○
Sinto-me satisfeito/a quando comunico/convivo à distância com familiares e amigos (chamadas, videochamadas, redes sociais)	○	○	○	○	○	○

(Adaptado de F-SozU K-6; Li et al, 2018)

15. **Até que ponto concorda** com cada uma das seguintes **afirmações** relativas ao seu **estilo de vida e atividades de lazer**, nesta altura:

	Não concordo de todo	Não concordo	Não sei ao certo	Concordo	Concordo totalmente	Não se aplica ao meu caso
Consigo manter os meus passatempos/*hobbies* habituais	O	O	O	O	O	O
Consigo manter uma rotina diária (hora de acordar e deitar, refeições, trabalho, etc.)	O	O	O	O	O	O
A atividade física que estou a fazer é importante para mim	O	O	O	O	O	O
A ajuda que estou a dar a outras pessoas (ações de voluntariado ou outras) é importante para mim	O	O	O	O	O	O
Assistir a eventos culturais *online* (teatro, óperas, exposições, concertos…) é importante para mim	O	O	O	O	O	O
Ler é importante para mim (livros, revistas, jornais)	O	O	O	O	O	O
Procurar informação e ver notícias sobre a pandemia é importante para mim	O	O	O	O	O	O
Dou por mim a procurar informação e a ver notícias sobre a pandemia ao longo de todo o dia.	O	O	O	O	O	O
Ver televisão, filmes, séries, documentários é importante para mim	O	O	O	O	O	O
Fazer jardinagem ou pequenas obras e outros trabalhos manuais é importante para mim	O	O	O	O	O	O
Jogar jogos (videojogos, jogos de tabuleiro, palavras-cruzadas, sudoku) é importante para mim	O	O	O	O	O	O
É difícil para mim ter deixado a minha prática religiosa em comunidade	O	O	O	O	O	O

É difícil para mim ter deixado de ir ao café, restaurantes, fazer compras e outras atividades (praias, caminhar, viagens, etc.)	○	○	○	○	○	○

ACESSO A SERVIÇOS E MEIOS DE PROTEÇÃO

16. **Até que ponto concorda** com cada uma das seguintes **afirmações** relativas ao **acesso aos serviços e cuidados de saúde**, nesta **altura da pandemia:**

	Não concordo de todo	Não concordo	Não sei ao certo	Concordo	Concordo totalmente	Não se aplica ao meu caso
Continuo a ter o mesmo acesso aos cuidados de saúde de que preciso, no Serviço Nacional de Saúde (centros de saúde, hospitais, ...)	○	○	○	○	○	○
Continuo a ter o mesmo acesso aos serviços de urgência	○	○	○	○	○	○
Continuo a ter o mesmo acesso aos cuidados de saúde de que preciso, no setor privado (hospitais privados, consultórios privados, ...), e setor social (hospitais, ...)	○	○	○	○	○	○
Tenho acesso fácil à linha SNS 24	○	○	○	○	○	○
Tenho acesso fácil a linhas de apoio psicológico e emocional	○	○	○	○	○	○
Tenho acesso fácil a apoio psicológico e emocional presencial	○	○	○	○	○	○
Tenho acesso fácil a cuidados de saúde domiciliários	○	○	○	○	○	○
Tenho acesso fácil a proteções (máscaras, luvas, álcool, desinfetantes, ...)	○	○	○	○	○	○

FASE DE DESCONFINAMENTO GRADUAL

17. **Até que ponto concorda** com cada uma das seguintes **afirmações** relativas ao **período de desconfinamento:**

	Não concordo de todo	Não concordo	Não sei ao certo	Concordo	Concordo totalmente
Preocupa-me que as medidas agora adotadas sejam insuficientes	○	○	○	○	○
Preocupa-me que o desconfinamento gradual coloque em maior risco as pessoas mais frágeis (p. ex. idosos)	○	○	○	○	○
Preocupa-me regressar ao trabalho, no local habitual	○	○	○	○	○
Preocupa-me as condições de proteção que vou encontrar quando regressar ao trabalho	○	○	○	○	○
Preocupa-me deslocar-me em transportes públicos	○	○	○	○	○
Preocupa-me ter de usar máscara durante todo o dia, no meu local de trabalho	○	○	○	○	○
Preocupa-me continuar desempregado e sem apoios financeiros	○	○	○	○	○
Preocupa-me que os outros não cumpram as medidas de distanciamento social, proteção e segurança recomendadas	○	○	○	○	○
Preocupa-me frequentar espaços comuns (restaurantes, cabeleireiros…)	○	○	○	○	○

APÓS A PANDEMIA

18. Até que ponto concorda com cada uma das seguintes **afirmações**, em relação ao **futuro depois da pandemia:**

	Não concordo de todo	Não concordo	Não sei ao certo	Concordo	Concordo totalmente
Preocupa-me não saber quando teremos um tratamento ou vacina eficazes	○	○	○	○	○
Preocupa-me a possibilidade de o país entrar numa crise económica muito grave	○	○	○	○	○
Preocupa-me não conseguir recuperar o rendimento financeiro que tinha antes da pandemia	○	○	○	○	○
Preocupa-me que a minha forma de viver não volte a ser a mesma	○	○	○	○	○
Algumas das formas alternativas de organização do trabalho utilizadas neste momento podem ser positivas (ex.: teletrabalho)	○	○	○	○	○
Sinto-me otimista em relação ao futuro	○	○	○	○	○

DADOS SOCIODEMOGRÁFICOS E PROFISSIONAIS

19. Sexo
O Feminino
O Masculino

20. Qual é a sua **data de nascimento?** ___ - ___ - __________

21. Qual a sua **nacionalidade?**
O Portuguesa
O Outra. Qual? ________

22. Qual o **grau de escolaridade mais elevado** que **atingiu?**
O Instrução primária incompleta
O 4º ano (instrução primária) completo
O 6º ano (2º ano liceal / 2º ciclo do ensino básico) completo
O 9º ano (5º ano liceal / 3º ciclo do ensino básico) completo
O 12º ano (ou 7º ano liceal / Ensino secundário) completo
O Licenciatura ou outro grau académico (estudos superiores)

23. **Por mês,** qual é o seu **rendimento médio líquido?** [Indique por favor o valor líquido que recebe a título individual (não o do agregado familiar), já depois dos impostos retidos.]
OMenos de 500 euros
OEntre 500 e 750 euros
OEntre 751 e 1000 euros
OEntre 1001 e 1250 euros
OEntre 1251 e 1500 euros
OEntre 1501 e 2000 euros
OEntre 2001 e 2500 euros
OEntre 2501 e 3000 euros
OMais de 3000 euros
OPrefiro não responder

24. Indique o seu código postal *[Indique apenas os 4 primeiros dígitos do código postal]*

_____

25. Qual a sua situação face ao emprego/trabalho?
O Empregado
    O Trabalho por conta própria
        _Mantenho a atividade profissional
        _Suspendi a atividade profissional temporariamente

○ Trabalho por conta de outrem
_Com contrato permanente
_Com contrato a termo
_Em regime de *lay-off*

○ Desempregado
_Antes da pandemia?
_Durante a pandemia?

○ Reformado
○ Estudante
○ Doméstica/o
○ Cuidador/a informal
○ Outra situação. Qual? ________

*Abre para quem respondeu "EMPREGADO" E ("Mantenho a atividade profissional" OU "Com contrato permanente" OU "Com contrato a termo")*26. Indique a forma **como trabalha nesta fase atual da pandemia**

○ Em casa, em teletrabalho
○ No local de trabalho, em contacto com o público
○ No local de trabalho, sem contacto com o público
○ Outra. Qual? ____________

Abre para quem respondeu "EMPREGADO" E ("Mantenho a atividade profissional" OU "Com contrato permanente" OU "Com contrato a termo") **27. Em que regime?**
○ Em regime normal (nas mesmas condições que tinha antes da pandemia)
○ Passei a estar em regime de rotação/turnos/espelho
○ Outra. Qual? ____________

Abre para quem respondeu "EMPREGADO" E ("Mantenho a atividade profissional" OU "Com contrato permanente" OU "Com contrato a termo") **28. Atendendo à sua <u>atividade profissional</u>**, teve **necessidade de se afastar da família com quem vive habitualmente**?
○ Sim
○ Não

Abre para quem respondeu "EMPREGADO" E ("Mantenho a atividade profissional" OU "Com contrato permanente" OU "Com contrato a termo") **29. Para muitas pessoas, o horário de trabalho pode ter sofrido alterações. No seu caso pessoal...**
○ Reduziu o horário de trabalho
○ Aumentou o horário de trabalho
○ Manteve o número de horas de trabalho

Abre para quem respondeu "EMPREGADO" **30. Qual das seguintes opções descreve a sua <u>profissão principal</u>**?

○ Profissional de saúde (médico/enfermeiro/farmacêutico/ técnico superior de diagnóstico e terapêutica/ técnico superior de saúde/administrador hospitalar/auxiliar de ação médica...)
○ Profissional de saúde dentária
○ Profissional de farmácias de comunidade
○ Profissional em lares de idosos
○ Bombeiro/Proteção civil/Militar
○ Profissional de segurança pública (PSP, GNR, segurança...)
○ Profissionais de serviços prisionais
○ Taxista/ Motorista de transportes públicos/TVDE
○ Comerciante/Lojista/Restauração e hotelaria/Cabeleireiro e estética (área de atendimento ao público)
○ Operário fabril
○ Carteiro/Estafeta
○ Profissional de educação (professor, auxiliar)
○ Outra. Qual? _______________

Abre para quem respondeu "PROFISSIONAL DE SAÚDE" 29. Indique, por favor, a sua carreira
○ Médico
○ Enfermeiro
○ Auxiliar de ação médica
○ Técnico Superior de Diagnóstico e Terapêutica
○ Técnico Superior de Saúde
○ Administrador hospitalar
○ Farmacêutico
○ Outra, qual? __________

Abre para quem respondeu "PROFISSIONAL DE SAÚDE" 30. Indique o setor principal
○ Público (inclui Parcerias Público-Privadas)
○ Privado
○ Social da Saúde

Abre para quem respondeu "Setor público" 31.a. Local onde desempenha funções
○ Hospital
○ Agrupamento de Centros de Saúde (ACES)
○ Serviço de Saúde Pública
○ Unidade Local de Saúde (ULS)
○ Instituto Nacional de Emergência Médica (INEM)
○ Unidades de Intervenção Local em Comportamentos Aditivos e Dependências (CAD)
○ Instituto Nacional de Saúde Doutor Ricardo Jorge
○ Outro, qual? _________________

Abre para quem respondeu "Setor privado" OU "Social da saúde" 31.a. Local onde desempenha funções

O Hospital

O Clínica/Casa de Saúde

O Consultório

O Laboratório

O Outro. Qual? _______________

Abre para quem respondeu "EMPREGADO" E ("Mantenho a atividade profissional" OU "Com contrato permanente" OU "Com contrato a termo") E ("Profissional de saúde" OU Profissional de saúde dentária" OU Profissional de farmácias de comunidade") **32. Continua a ter contacto regular e presencial com doentes?**

O Não

O Sim

O Não se aplica

Abre para quem respondeu "EMPREGADO" E ("Mantenho a atividade profissional" OU "Com contrato permanente" OU "Com contrato a termo") E ("Profissional de saúde") **33. Está na 'linha da frente', ou seja, a tratar pessoas infetadas com COVID-19?**

O Não

O Sim

O Não se aplica

Abre para quem respondeu "EMPREGADO" E ("Mantenho a atividade profissional" OU "Com contrato permanente" OU "Com contrato a termo") E ("Profissional de saúde" OU "Investigadores e outros técnicos ligados à investigação no âmbito da COVID-19") **34. Está na 'linha da frente' a fazer diagnóstico laboratorial de SARS-Cov-2?**

O Não

O Sim

O Não se aplica

Abre para quem respondeu "EMPREGADO" E ("Mantenho a atividade profissional" OU "Com contrato permanente" OU "Com contrato a termo") E ("Profissional em lares de idosos" OU "Bombeiro/Proteção civil/Militar" OU "Profissionais de serviços prisionais") **35. Teve que contactar/cuidar de doentes com COVID-19 no âmbito da sua atividade profissional?**

O Não

O Sim

O Não tenho a certeza

Abre para quem respondeu "EMPREGADO" E ("Mantenho a atividade profissional" OU "Com contrato permanente" OU "Com contrato a termo") E Qualquer uma das profissões **36. Durante as últimas 4 semanas, como descreve o seu desempenho geral no trabalho, nos dias em que trabalhou?** (a nota 0 representa o pior desempenho no trabalho que alguém poderia ter na sua função, e a nota 10 representa o desempenho do melhor trabalhador nesta mesma função)

0 10

Abre para quem respondeu "EMPREGADO" E ("Mantenho a atividade profissional" OU "Com contrato permanente" OU "Com contrato a termo") E Qualquer uma das profissões **37. Como se sente no seu trabalho?**

Abaixo vai encontrar um conjunto de afirmações que descrevem diferentes **sentimentos** que pode ter relativamente ao seu trabalho.
Por favor, indique quantas vezes nos últimos **30 dias** (1 mês) se sentiu assim no seu trabalho.

	Nunca ou quase nunca	Muito poucas vezes	Poucas vezes	Por vezes	Algumas vezes	Muitas vezes	Sempre ou quase sempre
Sinto-me cansado/a							
Ao acordar, sinto-me sem energia para ir trabalhar							
Sinto-me fisicamente esgotado/a							
Sinto-me fatigado/a de trabalhar							
Sinto-me como se estivesse sem "bateria"							
Sinto-me sem forças							
Sinto lentidão na minha capacidade de pensar							
Tenho dificuldades em concentrar-me							
Sinto que não consigo pensar com clareza							
Sinto que não consigo concentrar-me no que penso							

Tenho dificuldades em pensar sobre coisas complexas/difíceis

Sinto-me incapaz de ser sensível às necessidades dos outros (p. ex., colegas de trabalho, clientes, etc.)

Sinto-me incapaz de ter uma boa relação com os outros (p. ex., colegas de trabalho, clientes, etc.)

Sinto-me incapaz de ser simpático/a com os outros (p. ex., colegas de trabalho, clientes, etc.)

(MBSM, Gomes, 2012)

37. Em que tipo de habitação vive, atualmente?

O Apartamento com uma assoalhada
O Apartamento com duas assoalhadas
O Apartamento com três ou mais assoalhadas
O Quarto ou parte de casa alugada
O Moradia/Vivenda
O Instituição/Lar
O Outra. Qual? ...

38. A habitação **tem espaço exterior (p. ex., varanda, terraço, jardim)**?

O Sim
O Não

39. Tem animal ou animais de companhia?

O Sim
O Não

40. Com quantas pessoas partilha habitação, neste momento?

O Vivo sozinho
O Vivo com mais pessoas. Quantas? _________

[Filtro1 para VIVO COM MAIS PESSOAS]

41. Qual a relação com a(s) pessoa(s) com quem está a partilhar habitação, neste momento? [opção múltipla]

❍ Cônjuge/namorado/a

❍ Filho(a)/s

❍ Pai/mãe

❍ Avô/Avó

❍ Colegas/amigos

❍ Outros _________

[Filtro2 para VIVO COM MAIS PESSOAS]

42. Qual a **faixa etária das pessoas com quem partilha habitação, neste momento?** [opção múltipla]

❍ 0-5 anos

❍ 6-12 anos

❍ 13-17 anos

❍ 18-29 anos

❍ 30-69 anos

❍ 70 ou mais anos

❍ Outra. Qual? ________

43. Como teve conhecimento deste questionário?

❍ Entidade empregadora (hospital, centro de saúde, outro tipo de unidade de saúde)

❍ Entidade empregadora (não se trata de uma unidade de saúde)

❍ Ordem profissional

❍ Redes sociais

❍ Página institucional

❍ Familiares/amigos

❍ Outra. Qual?

As suas respostas foram submetidas com sucesso.

Gostaríamos de contar com a sua colaboração no preenchimento de outro questionário, numa fase posterior da pandemia. Se autorizar, clique em "Seguinte" e indique-nos um contacto de email para onde possamos enviar-lhe o convite para esse questionário.

Se não concordar com esse contacto posterior, aceite, desde já, o nosso muito obrigado pela sua colaboração!

Por favor, indique-nos o endereço de email através do qual o podemos contactar para esse efeito.

De forma a podermos fazer a correspondência entre este questionário e outro que venha a responder, agradecemos que repita aqui a sua data de nascimento

____

Para o mesmo fim, agradecemos também que repita aqui o seu código postal (Indique apenas os 4 primeiros dígitos do código postal)

____

Obrigado pela sua participação!
